# Heart failure in obesity: insights from proteomics in patients treated with or without weight-loss surgery

**DOI:** 10.1038/s41366-022-01194-0

**Published:** 2022-08-09

**Authors:** Kristjan Karason, Nicolas Girerd, Johanna Andersson-Asssarsson, Kevin Duarte, Magdalena Taube, Per-Arne Svensson, Anne-Cecile Huby, Markku Peltonen, Lena M. Carlsson, Faiez Zannad

**Affiliations:** 1 Department of Cardiology and Transplant Institute, Sahlgrenska University Hospital, Gothenburg, Sweden; 2 Department of Molecular and Clinical Medicine, Institute of Medicine, Sahlgrenska Academy, University of Gothenburg, Gothenburg, Sweden; 3 Centre d’Investigation Clinique 1433 module Plurithématique, CHRU Nancy—Hopitaux de Brabois, Institut Lorrain du Coeur et des Vaisseaux Louis Mathieu, Vandoeuvre les Nancy, France; 4 Institute of Health and Care Sciences, Sahlgrenska Academy at University of Gothenburg, Gothenburg, Sweden; 5 National Institute for Health and Welfare, Helsinki, Finland

**Keywords:** Translational research, Cardiovascular diseases

## Abstract

**Background:**

Obesity is associated with incident heart failure (HF), but the underlying mechanisms are unclear.

**Methods:**

We performed a nested case-control study within the Swedish-Obese-Subjects study, by identifying 411 cases who developed HF and matched them with respect to age, sex, weight-loss-surgery and length of follow-up with 410 controls who did not develop HF. In analyses corrected for multiple testing, we studied 182 plasma proteins known to be related to cardiovascular disease to investigate whether they could add to the understanding of the processes underlying obesity-related HF.

**Results:**

A total of 821 subjects were followed for 16 ± 6 years. Multivariable analysis adjusted for matching variables revealed that 32 proteins were significantly associated with HF. Twelve proteins were related to HF ≥ 80% of the time using a bootstrap resampling approach (false-discovery-rate [FDR] < 0.05): 11 were associated with increased HF-risk: TNFRSF10A*, ST6GAL1, PRCP, MMP12, TIMP1, CCL3, QPCT, ANG, C1QTNF1, SERPINA5 and GAL-9; and one was related to reduced HF-risk: LPL. An further 20 proteins were associated with onset of HF 50–80% of the time using bootstrap resampling (FDR < 0.05). A pathway analysis including all significant 32 proteins suggested that these biomarkers were related to inflammation, matrix remodeling, cardiometabolic hormones and hemostasis. Three proteins, C1QTNF1, FGF-21 and CST3, reflecting dyslipidemia and kidney disease, displayed a higher association with HF in patients who did not undergo weight-loss-surgery and maintained with obesity.

**Conclusion:**

Pathways associated with HF in obesity include inflammation, matrix remodeling, cardiometabolic hormones and hemostasis; three protein biomarkers predicting HF appeared to be obesity-specific.

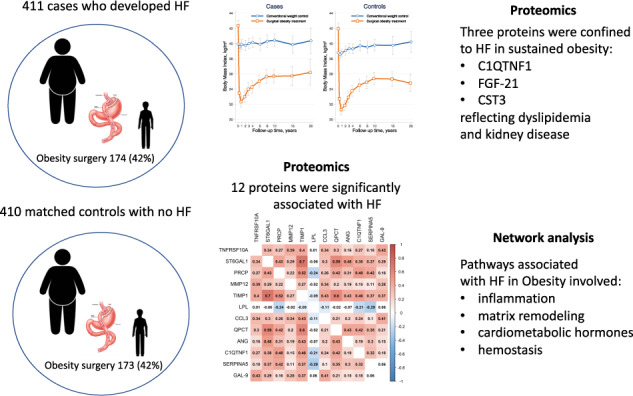

## Introduction

Obesity, which is widely prevalent in many European countries [1] and the USA [2], has repeatedly been shown to be associated with heart failure (HF) [3, 4]. Weight control is recommended to optimize HF risk factors but weight reduction is difficult to achieve with conventional lifestyle changes, and the results are often not sustained. By contrast, bariatric surgery is an effective and safe treatment option that results in large weight losses maintained over time [5]. The Swedish Obese Subjects (SOS) study is an ongoing controlled intervention trial that compares the effects of bariatric surgery and usual obesity care on morbidity and mortality during long-term follow-up [6].

Various risk models for HF development have been advanced [7, 8], but little is known currently about the mechanistic pathways that link obesity with the development of HF. Proteomics has emerged as a powerful tool to explore the underlying mechanisms of HF development in people with obesity [9–11]. By applying a network analysis, involving established protein-protein interaction data, various links among protein biomarkers (BMs) of pathophysiological importance can be described. Matching obesity preventive strategies to mechanistic biotargets offers the possibility of more effective reduction of HF incidence.

The aim of the present study was to investigate whether proteomic data may add to the understanding of the mechanisms underlying obesity-related development of HF during long-term follow-up in the SOS study. Furthermore, we studied whether BMs related to heart failure differed between patients who lost weight through surgery, as opposed to those who maintained with obesity after usual care.

## Methods

### SOS study

The ongoing prospective controlled SOS intervention study (ClinicalTrials.gov identifier: NCT02556450) comparing the effects of weight loss by means of bariatric surgery versus usual obesity care during long-term follow-up has previously been described in detail [12]. In brief, 4047 participants aged 37–60 years with obesity (BMI ≥ 34 kg/m^2^ for men and ≥38 kg/m^2^ for women) were enrolled between 1 September 1987 and 31 January 2001. The intervention group included 2010 individuals who had expressed a preference for treatment with bariatric surgery. A reference group of 2037 participants who received non-surgical treatments was created using an automatic matching program. The SOS study was approved by seven Swedish ethical review boards and all participants gave written or oral consent.

### Obesity intervention

The surgical procedures used in the SOS study were vertical banded gastroplasty (68%), gastric banding (19%) and gastric bypass (13%) [5]. The conventional treatment offered to subjects in the non-surgical group was not predefined but instead adhered to local routines at the primary healthcare centers.

### Outcome of the study

The outcome of this case-control study was first-time detection of HF as a principal diagnosis. This was accomplished by cross-checking the SOS database with the Swedish National Patient Register and the Swedish Cause of Death Register for the following diagnosis codes: 428 (International Classification of Diseases-9 until 1996) and I50 (International Classification of Diseases-10 from 1997).

### Study subjects

We employed a nested matched case-control design in which individuals who developed HF after inclusion in the SOS were considered to be at risk (i.e. eligible to be selected as a control up until the time at which they became a case) [13]. For each case we aimed to identify a corresponding control matched with respect to age, sex, weight-loss surgery and follow-up time.

### Sampling and biomarkers

Plasma (from tubes coated with ethylenediaminetetraacetic acid [EDTA]) was collected at baseline in the morning after an overnight fast and stored at –80 °C until evaluation. After thawing, the plasma was transferred to TATAA Biocentre (Gothenburg, Sweden) for analysis, which was performed in a blinded fashion. Protein expression was determined with the Proseek® Multiplex kit technique (Olink Bioscience, Uppsala, Sweden) using the Cardiovascular II and Cardiometabolic panels, each of which determines 92 protein BMs.

The two platforms, described in detail elsewhere (www.olink.com), provide log_2_-normalized protein expression data. The limit of detection (LOD) was defined as three standard deviations above background and reported in picograms per milliliter for all assays in which recombinant protein antigen was available. Protein BMs with >80% of values below the LOD were excluded from the analysis, which led to the omission of six proteins. The abbreviations, full names and respective Olink® multiplex panels of all proteins (proteins studied and excluded) are described in the Supplementary Table 1.

### Statistical analysis

All analyses were performed using R software version 4.0.0 (The R Project for Statistical Computing; https://www.r-project.org/). Continuous variables are presented as mean ± standard deviation and categorical variables as numbers with percentages in parentheses. Patients’ baseline characteristics were compared between cases and controls using t-tests for continuous variables and Fisher’s exact test for categorical variables.

A power analysis was performed based on the obtained sample size and parameter estimates [14]. For 821 patients, an overall probability of 50% to develop HF and by using a logistic regression to evaluate association between incident HF (binary response) and one biomarker (continuous variable) assumed to be normally distributed, a standardized odds-ratio (sOR) ≥1.27 or sOR ≤0.79 allowed us to reach a power of at least 80% at a 1% alpha significance level.

Logistic regression models adjusted for the matching variables were used to identify candidate protein BMs associated with development of HF. Multiple testing was corrected for by using a false discovery rate (FDR) of 5% applying the Benjamini–Hochberg procedure [15]. A bootstrap sampling approach was applied to rank the most essential proteins (5000 bootstraps). Models were fitted for each bootstrap sample and we studied the proportion of times each BM was significant using an FDR of 5%. Proteins associated with incident HF for ≥80% of the time according to the bootstrap resampling approach were considered of major mechanistic importance, whereas those associated with incident HF for 50–80% of the time were considered to be of potential significance.

### Network analysis

We used a pathway analysis with an induced-network approach facilitated by the ConsensusPathDB online server (accessed in May 2020) at the Max Planck Institute for Molecular Genetics (Berlin, Germany) to identify the links among the 32 proteins associated with HF > 50% of the time according to the resampling method (with adjustment for the matching variables) and based on knowledge of interaction networks (protein, genetic, biochemical and gene-regulatory interactions) [16].

### Interactions between BMs and obesity intervention

Interaction between BMs (HF > 50% of the time according to resampling and FDR < 5%) and obesity intervention with respect to HF development was assessed using logistic model adjusting for matching variables (age, sex, bariatric surgery and time to incident HF among cases). Given the low power of interaction tests a significance level of 0.10 was used for interaction *p* values [17, 18].

## Results

### Baseline characteristics and changes in weight

**Fig. 1:**
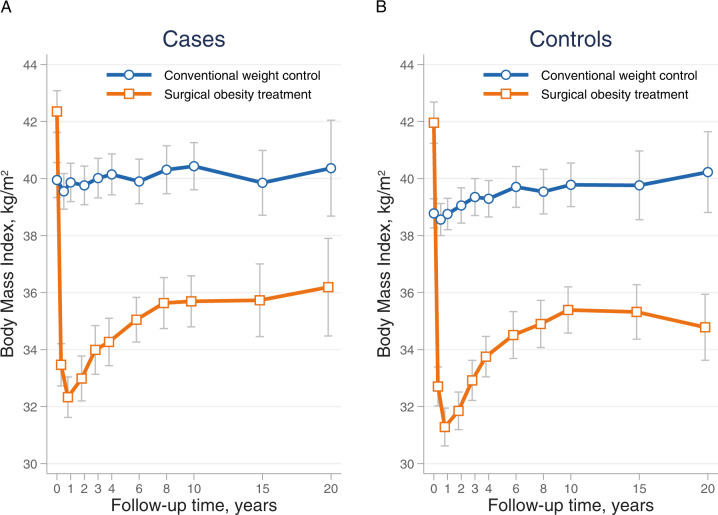
Changes in BMI in the two study groups during follow-up. Changes in BMI over time for **A** cases (*n* = 411) and **B** matched controls (*n* = 410) treated with and without bariatric surgery, respectively.

Of the 4037 participants in the SOS study without HF at baseline, we identified 411 patients with incident HF. Due to a missing plasma sample, the matching procedure generated a total of 410 controls. These 821 participants had a mean duration of follow-up of 15.6 ± 5.9 years. Weight-loss surgery was performed in 42% of patients. Although the cases and controls were well matched with regard to age, sex, bariatric surgery and time to incident HF, cases had a greater BMI, a higher heart rate and, in general, a more unpropitious cardiovascular risk profile than the controls (Table 1). Changes in BMI over time for cases and matched controls treated with and without bariatric surgery are shown in Fig. 1.

**Table 1:** Baseline characteristics of the study population^a^.

Characteristic	Cases (*n* = 411)	Controls (*n* = 410)	*p* value
Age (years)	51.0 ± 6.0	51.0 ± 6.0	0.98
Males	188 (45.7)	188 (45.9)	1.00
Bariatric surgery	174 (42.3)	173 (42.2)	0.99
Duration of incident HF (years)	15.6 ± 5.9	15.6 ± 5.9	0.98
Body mass index (kg/m^2^)	41.0 ± 5.0	40.1 ± 4.6	0.011
Systolic blood pressure (mmHg)	145.9 ± 19.0	142.6 ± 18.3	0.011
Diastolic blood pressure (mmHg)	89.5 ± 11.3	89.1 ± 10.9	0.54
Heart rate (beats/min)	71.8 ± 13.8	69.3 ± 12.6	0.007
Creatinine (µmol/l)	70.6 ± 11.0	70.9 ± 9.6	0.69
Total cholesterol (mmol/l)	5.95 ± 1.12	5.83 ± 1.11	0.11
HDL cholesterol (mmol/l)	1.31 ± 0.33	1.38 ± 0.36	0.008
Triglycerides (mmol/l)	2.41 ± 1.71	2.07 ± 1.53	0.002
Blood glucose (mmol/l)	5.45 ± 2.19	4.99 ± 1.73	0.001
Diabetes	92 (22.4)	64 (15.6)	0.016
Smoking status			0.011
Former smoker	179 (43.6)	177 (43.2)	
Current smoker	104 (25.3)	73 (17.8)	
Never smoker	128 (31.1)	160 (39.0)	

^a^Values are presented in the form mean ± standard deviation or frequency (percentage).

*HDL* high-density cholesterol, *HF* heart failure.

### Biomarkers associated with incident HF

An analysis adjusted for the matching variables of age, sex, bariatric surgery and time to follow-up time among cases revealed that 32 proteins were significantly associated with incident HF: 12 were retained ≥80% of the time according to the bootstrap resampling approach using FDR of <5% and a further 20 proteins were associated with onset of HF between 50 and 80% of the time using the same bootstrap resampling approach (Table 2). Association with incident HF for all studied proteins are reported in Supplementary Table 2.

**Table 2:** Protein biomarkers associated with incident HF using logistic regression models adjusted for the matching variables (age, gender, bariatric surgery and duration of incident HF) and assessment of the interaction with bariatric surgery.

Protein name	Panel	Global effect				Effect in patients without surgery		Effect in patients with surgery		*p* value for interaction^a^
		OR (CI 95 %)	*p* value	FDR adjusted p-value	Proportion of significant FDR adjusted p-values (bootstrap resampling)	OR (CI 95 %)	*p* value	OR (CI 95 %)	*p* value	
TNFRSF10A	CVD II	2.85 (1.86–4.37)	<0.0001	0.0002	98.6	2.92 (1.64–5.20)	0.0002	2.76 (1.47–5.18)	0.001	0.89
ST6GAL1	CM	2.29 (1.55–3.37)	<0.0001	0.002	93.2	2.51 (1.52–4.13)	0.0003	1.99 (1.08–3.65)	0.027	0.56
PRCP	CM	3.26 (1.75–6.06)	0.0001	0.007	89.1	3.65 (1.49–8.92)	0.004	2.93 (1.26–6.85)	0.012	0.72
MMP12	CVD II	1.53 (1.23–1.90)	0.0001	0.007	88.9	1.59 (1.20–2.12)	0.001	1.45 (1.05–2.00)	0.025	0.66
TIMP1	CM	1.97 (1.38–2.81)	0.0001	0.007	86.4	2.43 (1.49–3.99)	0.0004	1.56 (0.95–2.58)	0.079	0.21
LPL	CVD II	0.64 (0.50–0.82)	0.0003	0.008	85.9	0.63 (0.46–0.86)	0.004	0.66 (0.46–0.93)	0.019	0.85
CCL3	CVD II	1.53 (1.21–1.93)	0.0004	0.008	85.2	1.67 (1.20–2.32)	0.002	1.39 (1.00–1.93)	0.049	0.44
QPCT	CM	3.37 (1.71–6.65)	0.0004	0.008	84.9	2.89 (1.21–6.91)	0.016	4.25 (1.42–12.73)	0.009	0.58
ANG	CM	1.77 (1.29–2.42)	0.0003	0.008	83.0	1.81 (1.21–2.71)	0.004	1.71 (1.06–2.78)	0.029	0.86
C1QTNF1	CM	1.47 (1.18–1.82)	0.0004	0.008	82.4	1.80 (1.33–2.44)	0.0001	1.16 (0.85–1.57)	0.36	0.044
SERPINA5	CM	1.79 (1.28–2.50)	0.0007	0.009	80.1	1.56 (1.01–2.41)	0.045	2.17 (1.28–3.67)	0.004	0.34
GAL-9	CVD II	2.19 (1.39–3.44)	0.0006	0.009	80.0	2.71 (1.50–4.87)	0.0009	1.61 (0.80–3.23)	0.18	0.25
KIM1	CVD II	1.37 (1.14–1.65)	0.0007	0.010	79.6	1.42 (1.11–1.82)	0.005	1.32 (1.01–1.71)	0.039	0.65
COL18A1	CM	2.02 (1.35–3.04)	0.0006	0.009	79.4	2.50 (1.46–4.26)	0.0007	1.50 (0.80–2.80)	0.20	0.22
TRAIL-R2	CVD II	2.01 (1.33–3.03)	0.0008	0.010	77.9	2.11 (1.22–3.65)	0.007	1.89 (1.04–3.46)	0.038	0.79
IL-1RA	CVD II	1.36 (1.13–1.63)	0.0009	0.010	76.9	1.31 (1.03–1.67)	0.026	1.42 (1.08–1.86)	0.012	0.68
ICAM1	CM	1.80 (1.27–2.56)	0.0009	0.010	75.4	2.05 (1.29–3.26)	0.002	1.52 (0.89–2.59)	0.12	0.40
MMP7	CVD II	1.26 (1.09–1.46)	0.002	0.020	70.8	1.22 (1.01–1.48)	0.034	1.31 (1.04–1.65)	0.022	0.65
IL6	CVD II	1.35 (1.11–1.63)	0.002	0.020	70.4	1.38 (1.08–1.76)	0.009	1.30 (0.96–1.75)	0.088	0.74
FGF-21	CVD II	1.20 (1.07–1.35)	0.002	0.021	69.9	1.39 (1.18–1.64)	0.0001	1.02 (0.86–1.21)	0.84	0.010
CES1	CM	1.25 (1.08–1.44)	0.003	0.024	68.4	1.35 (1.10–1.65)	0.004	1.15 (0.93–1.42)	0.20	0.28
SCF	CVD II	0.64 (0.48–0.86)	0.003	0.024	68.2	0.59 (0.40–0.88)	0.010	0.70 (0.46–1.08)	0.10	0.57
PRSS8	CVD II	1.77 (1.21–2.60)	0.003	0.024	65.6	1.87 (1.16–3.03)	0.010	1.62 (0.89–2.96)	0.11	0.70
CST3	CM	1.69 (1.19–2.40)	0.003	0.024	65.3	2.26 (1.41–3.60)	0.0006	1.14 (0.67–1.94)	0.62	0.057
LILRB1	CM	1.82 (1.22–2.72)	0.003	0.025	64.7	2.09 (1.23–3.55)	0.006	1.50 (0.81–2.78)	0.19	0.42
REG1A	CM	1.46 (1.12–1.89)	0.004	0.029	62.7	1.36 (0.94–1.97)	0.10	1.56 (1.08–2.26)	0.017	0.60
NID1	CM	1.65 (1.17–2.34)	0.004	0.029	62.6	1.94 (1.20–3.13)	0.006	1.38 (0.84–2.28)	0.20	0.33
C2	CM	1.65 (1.17–2.32)	0.004	0.029	62.5	1.69 (1.09–2.63)	0.019	1.58 (0.92–2.71)	0.097	0.84
CFHR5	CM	1.54 (1.14–2.10)	0.005	0.033	61.2	1.98 (1.29–3.05)	0.001	1.19 (0.77–1.83)	0.43	0.10
IGLC2	CM	1.45 (1.11–1.90)	0.006	0.038	57.8	1.79 (1.23–2.62)	0.002	1.16 (0.79–1.69)	0.45	0.10
TCN2	CM	1.61 (1.13–2.29)	0.008	0.046	55.3	1.80 (1.12–2.87)	0.014	1.39 (0.81–2.37)	0.22	0.47
TIE1	CM	1.99 (1.19–3.31)	0.008	0.046	55.2	2.24 (1.11–4.52)	0.024	1.73 (0.83–3.64)	0.14	0.62

^a^Significant interactions with bariatric surgery were found for the following biomarkers: C1QTNF1, FGF-21 and CST3 (*p* < 0.10).

*CI* confidence interval, *CM* cardiometabolic, *CVD I* cardiovascular II, *FDR* false discovery rate, *HF* heart failure, *OR* odds ratio.

**Fig. 2:**
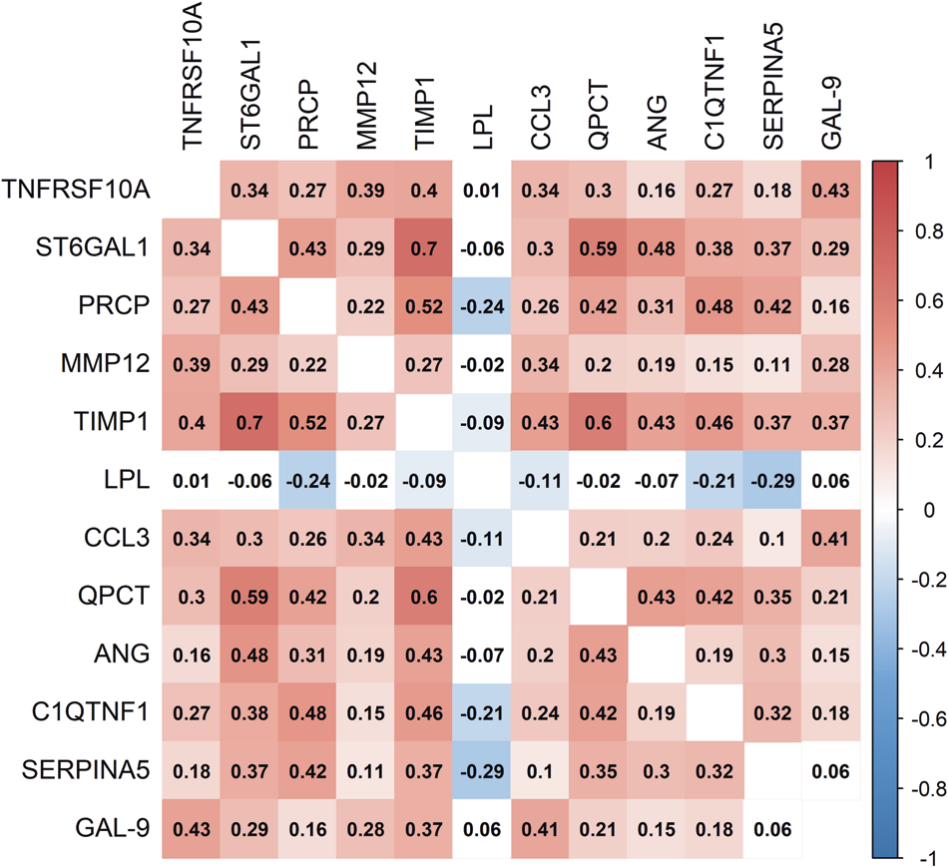
A correlation heatmap depicting the strength of relationships between protein biomarkers and incident HF. A heatmap based on Spearman correlations between the 12 essential protein BMs associated with HF development with an FDR < 5% and retained during 80% or more of the time according to the bootstrap resampling approach resampling approach.

Among the 12 proteins with the strongest association with HF, 11 were associated with increased risk of HF: TNFRSF10A, ST6GAL1, PRCP, MMP12, TIMP1, CCL3, QPCT, ANG, C1QTNF1, SERPINA5 and GAL-9; and one was related to reduced risk of HF: LPL (see Supplementary Table 1 for the full names of these proteins). A heatmap based on Spearman correlations between the 12 essential protein BMs associated with development of HF is shown in Fig. 2; a similar illustration for the 32 proteins associated with incident HF in the bootstrap approach for >50% of the time is shown in Supplementary Fig. 1.

### Network analysis

**Fig. 3:**
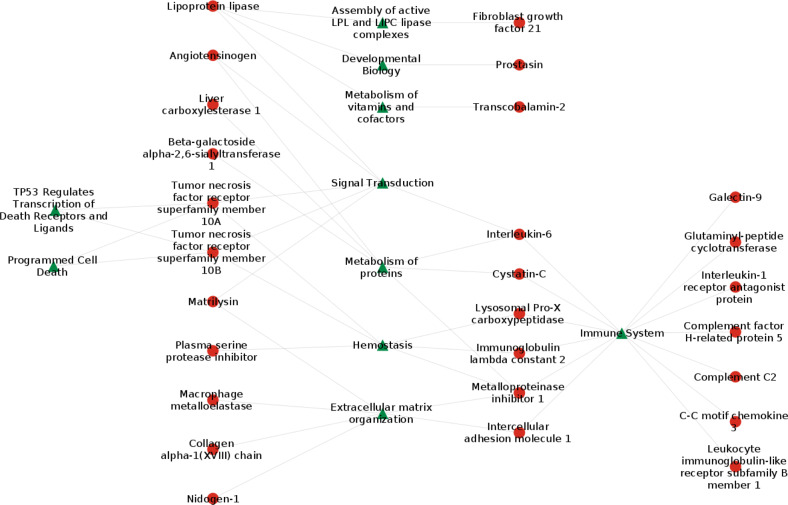
A network analysis illustrating the links among proteins associated with heart failure. A network analysis based on 32 proteins associated with HF more than 50% of the time according to the resample approach.

The network analysis based on the 32 proteins identified above revealed several underlying pathophysiological pathways that were important with respect to the development of HF in obesity (Fig. 3). These were mainly related to inflammation (cell surface interaction, interleukin signaling, features of the complement cascade, neutrophil degranulation), matrix remodeling (features related to metalloproteinase and collagen turnover), cardiometabolic hormones (angiotensin system and insulin-like growth factor) and hemostasis (PRCP and SERPINA5).

### Interactions between BMs and obesity intervention

In a heterogeneity analysis for the 32 candidate proteins, models adjusted for matching variables and clinical risk factors showed significant interactions of C1QTNF, FGF-21 and CST3 with obesity intervention (*p* < 0.10 for all). These three plasma proteins showed a statistically stronger association with onset of HF among patients who remained with obesity (after receiving usual care) compared with those who lost weight (after surgical intervention) (Fig. 1. and Table 2).

## Discussion

We have identified 12 plasma proteins that were significantly and strongly associated with incident HF in patients with obesity treated either with bariatric surgery or usual care (FDR < 0.05 and selected >80% of the time by our resampling method). Eleven of those proteins were associated with an increased risk of HF and one (LPL) was related to a lower risk.

### Biomarkers associated with incident HF and mechanisms revealed by network analysis

Among the 11 proteins that displayed a positive association with HF risk, PRCP has previously been linked to obesity [19] and/or diabetes [20]. Other BMs revealed to be associated with obesity-related HF are suggestive of mechanisms associated with apoptosis (TNFRSF10A) [21], inflammation (ST6GAL1 and GAL-9) [22] and fibrosis (MMP12, TIMP1 and QPCT) [23, 24]. Other proteins associated with incident HF in our research have been linked to coronary heart disease (ANG, C1QTNF1 and CCL3) [25] and thromboembolism (SERPINA5) [26] with the potential to induce myocardial damage [27, 28]. Conversely, LPL, the only protein in our investigation to show a negative relationship with the development of HF, has been associated with less severe atherosclerosis [29] and reduced rates of cardiovascular disease and death [30].

### Network analysis

The network analysis provided additional insight into how 32 proteins we identified as being associated with progression to HF may cluster into potential integrative mechanistic pathways (Fig. 3). Inflammation was featured by the expression of proteins mediating immune and inflammatory responses, as well as pathways stimulating complement activation. We also identified a cluster of proteins involved in apoptosis (TNFRSF10A and TNFRSF10B) and turnover of collagen fibrils and/or extracellular matrix organization.

In addition, we identified clusters of proteins more closely related to metabolic pathways including LPL, which appeared as a central node reducing the risk of HF. LPL was linked to fibroblast growth factor 21, a regulator of glucose homeostasis, to metabolizing vitamins and cofactors (transcobolamin-2) and to angiotensinogen, a potent controller of blood pressure, body fluid and electrolyte balance.

Finally, we found proteins related to atherosclerosis and aberrant hemostasis, including PRCP and SERPINA5. Circulating levels of PRCP have been found to reflect maladaptive biological processes related to obesity, diabetes, inflammation and atherosclerosis [20]. Serpinina5 has been suggested to be a regulator of hemostasis acting as a procoagulant and proinflammatory factor by inhibiting the anticoagulant activated protein C factor [31]. Elevated plasma concentrations of active SERPINA5 have been observed in survivors of myocardial infarction, and appear to represent a risk marker for acute coronary events [32].

### Interactions between BMs and obesity intervention

In participants who mostly remained with obesity despite conventional (non-surgical) anti-obesity measures, C1QTNF1, FGF-21 and CST3 were associated with a significantly higher risk of HF compared with those who lost weight through surgical intervention.

C1QTNF1 has been shown to be linked with dysregulation of lipid metabolism and an inflammatory response in macrophages [33], leading to coronary artery disease [34]. This suggests that early treatment with lipid lowering drugs such as statins may prevent CV diseases including HF in the population with sustained obesity.

FGF-21is predominantly expressed in the liver and adipose tissue and enhances glucose uptake by upregulation of glucose transporter-1, thereby improving glucose tolerance [35]. It has been suggested that obesity may cause reduced FGF-21sensitivity [36]. Surgically-induced weight loss might, therefore, be expected to restore FGF-21 sensitivity.

CST3 is a marker of renal function and a strong risk factor for HF and adverse cardiovascular events [37]. As bariatric surgery has been shown to slow decline in kidney function [38, 39], patients who do not undergo surgery and who retain excess body fat may be at increased risk of developing impaired kidney function, leading to HF, and in turn to further worsening of renal function. This creates an a priori case for using drugs that attenuate deterioration of renal function e.g. as ACE-inhibitors, angiotensin receptor blockers [40] and/or SGLT2 inhibitors [41] to reduce HF incidence in patients with persistent obesity

### Limitations

Limitations of the present study should be acknowledged. First, as a case-control study, this research identified protein BMs indicating risk of HF but cannot give any insights into causality. Second, the diagnosis of HF was determined by cross-linking the SOS database with the National Patient Register on the basis of inpatient and outpatient diagnosis codes, and with the Swedish Cause of Death Register, with a risk of missing cases. We are reassured, however, that ascertainment of HF as a principal diagnosis using the National Patient Register has a validity of 95% [42] and by the fact that the Cause of Death Register covers 99% of deaths in the Swedish population [43]. Third, we did not have access to ejection fraction (EF) data and were, therefore, unable to assess the potential value of the BMs with respect to HF with reduced or preserved EF. Fourth, due to the strong co-linearity between bariatric surgery and weight loss it is difficult disentangle their respective influences in statistical models. Fifth, the proteomics assay did not provide standard concentration units, making comparisons with clinically applied cut-offs difficult. We take reassurance, however, from the fact that the Olink standard procedures offer a high-quality multiplex protein quantification application encompassing high specificity, high sensitivity, and low sample consumption [44]. The choice of panels was based on proteins that have previously been associated with cardiovascular disease (Cardiovascular II and Cardiometabolic). Finally, a replication study involving prospective validation of these BMs in other populations with obesity is required to improve the external validity of these results.

## Conclusions

Progression towards HF in patients with obesity likely involves the interplay between several pathophysiological mechanisms, including inflammation, matrix remodeling, the cardiometabolic hormones and hemostasis. In a population with obesity, 12 proteins retained significant associations with incident HF after adjusting for common risk factors. Importantly, the protective role of LPL appears to be a key node in the mechanistic framework underlying onset of HF in network analysis. Three proteins (C1QTNF1, FGF-21 and CST3) were associated with incident HF among patients without bariatric surgery who retained excess body fat, suggesting that dyslipidemia and chronic kidney disease may constitute therapeutic targets to prevent the development of HF among patients with obesity.

## Supplementary information

Supplementary legends

Supplementary Figure 1

Supplementary Figure 2

Supplmentary Table 1

## Data Availability

The data that support the findings of this study are available from the corresponding author, KK, upon reasonable request
