# Supplementary material for: Heart failure in obesity: insights from proteomics in patients treated with or without weight-loss surgery
Source: Int J Obes (Lond). 2022 Aug 9;46(12):2088–94. doi: 10.1038/s41366-022-01194-0 (PMC9678794; doi:10.1038/s41366-022-01194-0)
Supplement: Supplementary file 1 — Supplementary legends [file 41366_2022_1194_MOESM1_ESM.docx]

# **Supplementary legends**

## Supplementary Figure 1. Heatmap including 32 proteins

A heatmap based on Spearman correlation between the 32 protein BMs associated with incident HF in the bootstrap approach above 50% of the time.

## Supplementary Table 1

Protein names and respective Olink® panel sorted in alphabetical order

## Supplementary Table 2

Association between proteins biomarkers and incident HF using logistic regression models adjusted for the matching variables (age, gender, bariatric surgery and duration of incident HF)

CI = confidence interval; CM = Cardiometabolic; CVD II = Cardiovascular II; FDR = false discovery rate; HF = heart failure; OR = odds ratio.
