# Supplementary material for: Heart failure in obesity: insights from proteomics in patients treated with or without weight-loss surgery
Source: Int J Obes (Lond). 2022 Aug 9;46(12):2088–94. doi: 10.1038/s41366-022-01194-0 (PMC9678794; doi:10.1038/s41366-022-01194-0)

Supplementary Figure 1

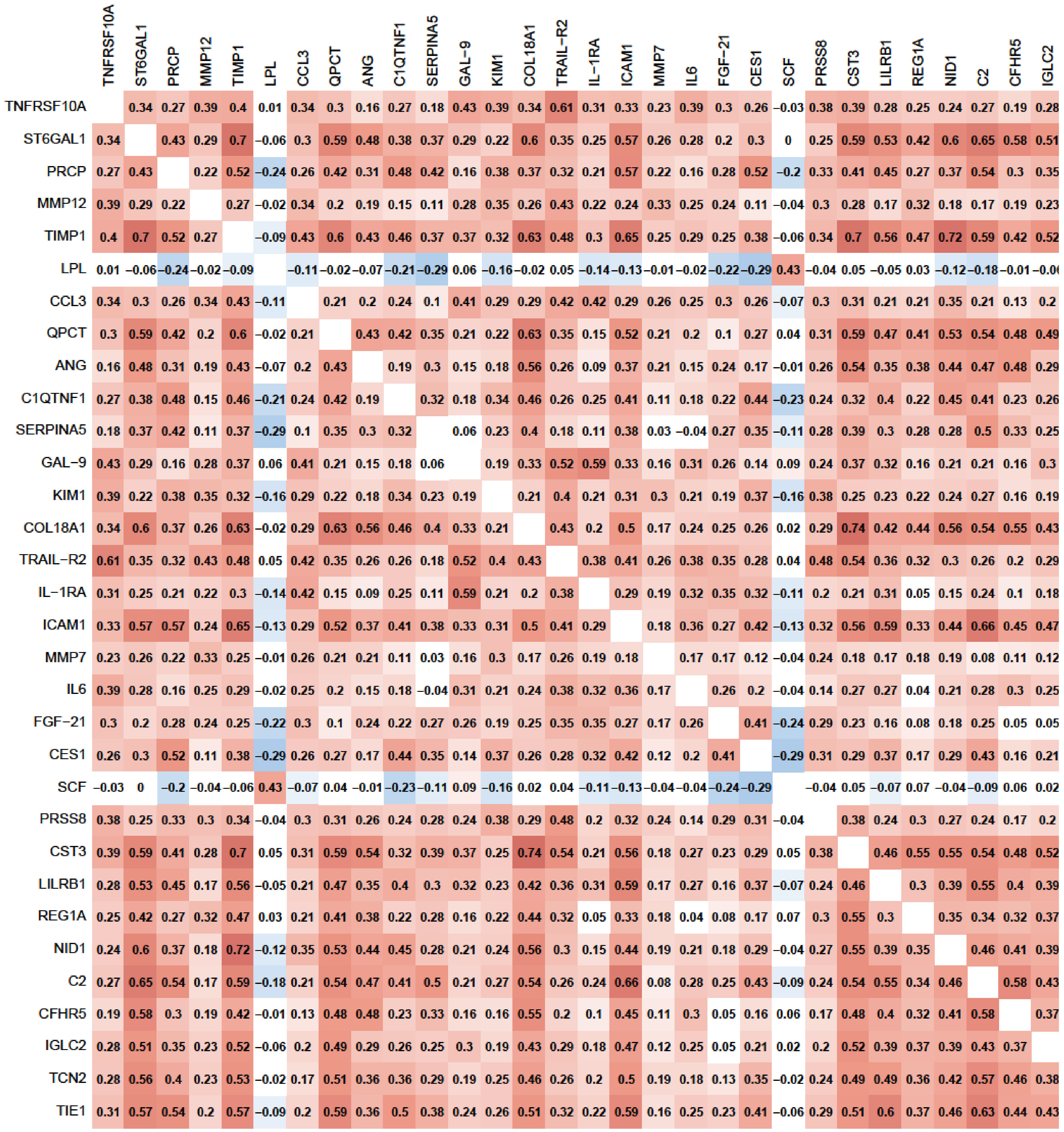

Supplement: Supplementary file 2 — Supplementary Figure 1 [file 41366_2022_1194_MOESM2_ESM.pdf]
