# Supplementary material for: Heart failure in obesity: insights from proteomics in patients treated with or without weight-loss surgery
Source: Int J Obes (Lond). 2022 Aug 9;46(12):2088–94. doi: 10.1038/s41366-022-01194-0 (PMC9678794; doi:10.1038/s41366-022-01194-0)
Supplement: Supplementary file 3 — Supplementary Figure 2 [file 41366_2022_1194_MOESM3_ESM.docx]

### Supplementary Table 1: Protein names and respective Olink® panel sorted in alphabetical order

| **Protein full name** | **Protein name** | **Panel** | **Uniprot ID** | **Proportion of values**  **below LOD (%)** | **Protein excluded from analysis** |
| --- | --- | --- | --- | --- | --- |
| Angiotensin-converting enzyme 2 | ACE2 | CVD II | Q9BYF1 | 0.0 |  |
| A disintegrin and metalloproteinase with thrombospondin motifs 13 | ADAM-TS13 | CVD II | Q76LX8 | 0.0 |  |
| ADM | ADM | CVD II | P35318 | 0.0 |  |
| Agouti-related protein | AGRP | CVD II | O00253 | 0.0 |  |
| Protein AMBP | AMBP | CVD II | P02760 | 0.0 |  |
| Angiogenin | ANG | CM | P03950 | 0.0 |  |
| Angiopoietin-1 | ANG-1 | CVD II | Q15389 | 0.0 |  |
| Angiopoietin-related protein 3 | ANGPTL3 | CM | Q9Y5C1 | 0.0 |  |
| Membrane primary amine oxidase | AOC3 | CM | Q16853 | 0.0 |  |
| Apolipoprotein M | APOM | CM | O95445 | 0.0 |  |
| Bone morphogenetic protein 6 | BMP-6 | CVD II | P22004 | 0.1 |  |
| Natriuretic peptides B | BNP | CVD II | P16860 | 88.8 | Excluded |
| Brother of CDO | BOC | CVD II | Q9BWV1 | 0.0 |  |
| Complement C1q tumor necrosis factor-related protein 1 | C1QTNF1 | CM | Q9BXJ1 | 0.0 |  |
| Complement C2 | C2 | CM | P06681 | 0.0 |  |
| Carbonic anhydrase 1 | CA1 | CM | P00915 | 0.0 |  |
| Carbonic anhydrase 3 | CA3 | CM | P07451 | 0.2 |  |
| Carbonic anhydrase 4 | CA4 | CM | P22748 | 0.0 |  |
| Carbonic anhydrase 5A, mitochondrial | CA5A | CVD II | P35218 | 2.9 |  |
| C-C motif chemokine 14 | CCL14 | CM | Q16627 | 0.0 |  |
| C-C motif chemokine 17 | CCL17 | CVD II | Q92583 | 0.0 |  |
| C-C motif chemokine 18 | CCL18 | CM | P55774 | 0.0 |  |
| C-C motif chemokine 3 | CCL3 | CVD II | P10147 | 0.0 |  |
| C-C motif chemokine 5 | CCL5 | CM | P13501 | 0.0 |  |
| T-cell surface glycoprotein CD4 | CD4 | CVD II | P01730 | 0.0 |  |
| CD40 ligand | CD40-L | CVD II | P29965 | 0.0 |  |
| Membrane cofactor protein | CD46 | CM | P15529 | 0.0 |  |
| CD59 glycoprotein | CD59 | CM | P13987 | 25.5 |  |
| SLAM family member 5 | CD84 | CVD II | Q9UIB8 | 0.0 |  |
| Cadherin-1 | CDH1 | CM | P12830 | 0.0 |  |
| Carcinoembryonic antigenrelated cell adhesion molecule 8 | CEACAM8 | CVD II | P31997 | 8.8 |  |
| Liver carboxylesterase 1 | CES1 | CM | P23141 | 0.5 |  |
| Complement factor H-related protein 5 | CFHR5 | CM | Q9BXR6 | 0.0 |  |
| Neural cell adhesion molecule L1-like protein | CHL1 | CM | O00533 | 0.0 |  |
| Beta-Ala-His dipeptidase | CNDP1 | CM | Q96KN2 | 0.0 |  |
| Collagen alpha-1 (XVIII) chain | COL18A1 | CM | P39060 | 0.0 |  |
| Cartilage oligomeric matrix protein | COMP | CM | P49747 | 0.0 |  |
| Complement receptor type 2 | CR2 | CM | P20023 | 0.0 |  |
| Cartilage acidic protein 1 | CRTAC1 | CM | Q9NQ79 | 0.0 |  |
| Cystatin-C | CST3 | CM | P01034 | 0.0 |  |
| Chymotrypsin C | CTRC | CVD II | Q99895 | 0.0 |  |
| Cathepsin L1 | CTSL1 | CVD II | P07711 | 0.0 |  |
| C-X-C motif chemokine 1 | CXCL1 | CVD II | P09341 | 0.0 |  |
| Decorin | DCN | CVD II | P07585 | 0.0 |  |
| 2,4-dienoyl-CoA reductase, mitochondrial | DECR1 | CVD II | Q16698 | 9.6 |  |
| Neutrophil defensin 1 | DEFA1 | CM | P59665 | 92.0 | Excluded |
| Dickkopf-related protein 1 | DKK-1 | CVD II | O94907 | 0.0 |  |
| Dipeptidyl peptidase 4 | DPP4 | CM | P27487 | 0.0 |  |
| EGF-containing fibulin-like extracellular matrix protein 1 | EFEMP1 | CM | Q12805 | 0.0 |  |
| Endoglin | ENG | CM | P17813 | 0.0 |  |
| Coagulation factor XI | F11 | CM | P03951 | 0.0 |  |
| Coagulation factor VII | F7 | CM | P08709 | 0.0 |  |
| Fatty acid-binding protein, intestinal | FABP2 | CVD II | P12104 | 0.0 |  |
| Prolyl endopeptidase FAP | FAP | CM | Q12884 | 39.2 |  |
| Low affinity immunoglobulin gamma Fc region receptor II-a | FCGR2A | CM | P12318 | 0.9 |  |
| Low affinity immunoglobulin gamma Fc region receptor III-B | FCGR3B | CM | O75015 | 0.0 |  |
| Ficolin-2 | FCN2 | CM | Q15485 | 0.0 |  |
| Fetuin-B | FETUB | CM | Q9UGM5 | 0.1 |  |
| Fibroblast growth factor 21 | FGF-21 | CVD II | Q9NSA1 | 0.0 |  |
| Fibroblast growth factor 23 | FGF-23 | CVD II | Q9GZV9 | 0.0 |  |
| Follistatin | FS | CVD II | P19883 | 0.0 |  |
| Galectin-9 | GAL-9 | CVD II | O00182 | 0.0 |  |
| Growth arrest-specific protein 6 | GAS6 | CM | Q14393 | 0.0 |  |
| Growth/differentiation factor 2 | GDF-2 | CVD II | Q9UK05 | 0.0 |  |
| Growth hormone | GH | CVD II | P01241 | 0.0 |  |
| Gastric intrinsic factor | GIF | CVD II | P27352 | 0.0 |  |
| Lactoylglutathione lyase | GLO1 | CVD II | Q04760 | 0.0 |  |
| Granulysin | GNLY | CM | P22749 | 16.2 |  |
| Platelet glycoprotein Ib alpha chain | GP1BA | CM | P07359 | 0.0 |  |
| Gastrotropin | GT | CVD II | P51161 | 0.2 |  |
| Hydroxyacid oxidase 1 | HAOX1 | CVD II | Q9UJM8 | 0.0 |  |
| Proheparin-binding EGF-like growth factor | HB-EGF | CVD II | Q99075 | 0.0 |  |
| Heme oxygenase 1 | HO-1 | CVD II | P09601 | 0.0 |  |
| Osteoclast-associated immunoglobulin-like receptor | HOSCAR | CVD II | Q8IYS5 | 0.0 |  |
| Heat shock 27 kDa protein | HSP-27 | CVD II | P04792 | 0.0 |  |
| Intercellular adhesion molecule 1 | ICAM1 | CM | P05362 | 0.0 |  |
| Intercellular adhesion molecule 3 | ICAM3 | CM | P32942 | 0.0 |  |
| Alpha-L-iduronidase | IDUA | CVD II | P35475 | 0.0 |  |
| Insulin-like growth factor-binding protein 3 | IGFBP3 | CM | P17936 | 0.0 |  |
| Insulin-like growth factor-binding protein 6 | IGFBP6 | CM | P24592 | 0.0 |  |
| Low affinity immunoglobulin gamma Fc region receptor II-b | IGG-FC-RECEPTOR-II-B | CVD II | P31994 | 0.0 |  |
| Ig lambda-2 chain C regions | IGLC2 | CM | P0CG05 | 0.0 |  |
| Pro-interleukin-16 | IL16 | CVD II | Q14005 | 0.0 |  |
| Interleukin-17D | IL-17D | CVD II | Q8TAD2 | 0.0 |  |
| Interleukin-18 | IL18 | CVD II | Q14116 | 0.0 |  |
| Interleukin-1 receptor antagonist protein | IL-1RA | CVD II | P18510 | 0.0 |  |
| Interleukin-1 receptor-like 2 | IL1RL2 | CVD II | Q9HB29 | 0.0 |  |
| Interleukin-27 | IL-27 | CVD II | Q8NEV9,Q14213 | 0.0 |  |
| Interleukin-4 receptor subunit alpha | IL-4RA | CVD II | P24394 | 1.1 |  |
| Interleukin-6 | IL6 | CVD II | P05231 | 0.0 |  |
| Interleukin-7 receptor subunit alpha | IL7R | CM | P16871 | 6.0 |  |
| Integrin alpha-M | ITGAM | CM | P11215 | 56.4 |  |
| Melusin | ITGB1BP2 | CVD II | Q9UKP3 | 24.8 |  |
| Kidney injury molecule 1 | KIM1 | CVD II | Q96D42 | 0.0 |  |
| Mast/stem cell growth factor receptor Kit | KIT | CM | P10721 | 0.0 |  |
| Neutrophil gelatinase-associated lipocalin | LCN2 | CM | P80188 | 61.8 |  |
| Leptin | LEP | CVD II | P41159 | 0.0 |  |
| Leukocyte immunoglobulin-like receptor subfamily B member 1 | LILRB1 | CM | Q8NHL6 | 0.0 |  |
| Leukocyte immunoglobulin-like receptor subfamily B member 2 | LILRB2 | CM | Q8N423 | 0.0 |  |
| Leukocyte immunoglobulin-like receptor subfamily B member 5 | LILRB5 | CM | O75023 | 0.0 |  |
| Lectin-like oxidized LDL receptor 1 | LOX-1 | CVD II | P78380 | 0.0 |  |
| Lipoprotein lipase | LPL | CVD II | P06858 | 0.0 |  |
| Latent-transforming growth factor beta-binding protein 2 | LTBP2 | CM | Q14767 | 82.6 | Excluded |
| Lymphatic vessel endothelial hyaluronic acid receptor 1 | LYVE1 | CM | Q9Y5Y7 | 0.0 |  |
| Macrophage receptor MARCO | MARCO | CVD II | Q9UEW3 | 0.0 |  |
| Mannose-binding protein C | MBL2 | CM | P11226 | 0.0 |  |
| Multiple epidermal growth factor-like domains protein 9 | MEGF9 | CM | Q9H1U4 | 0.0 |  |
| Tyrosine-protein kinase Mer | MERTK | CVD II | Q12866 | 0.0 |  |
| Hepatocyte growth factor receptor | MET | CM | P08581 | 0.0 |  |
| Microfibrillar-associated protein 5 | MFAP5 | CM | Q13361 | 0.0 |  |
| Matrix metalloproteinase-12 | MMP12 | CVD II | P39900 | 0.0 |  |
| Matrix metalloproteinase-7 | MMP7 | CVD II | P09237 | 0.0 |  |
| Neural cell adhesion molecule 1 | NCAM1 | CM | P13591 | 0.0 |  |
| NF-kappa-B essential modulator | NEMO | CVD II | Q9Y6K9 | 0.1 |  |
| Nidogen-1 | NID1 | CM | P14543 | 0.0 |  |
| Neurogenic locus notch homolog protein 1 | NOTCH1 | CM | P46531 | 0.0 |  |
| Neuropilin-1 | NRP1 | CM | O14786 | 0.0 |  |
| Oncostatin-M-specific receptor subunit beta | OSMR | CM | Q99650 | 1.0 |  |
| Peptidyl-glycine alpha-amidating monooxygenase | PAM | CM | P19021 | 0.0 |  |
| Pappalysin-1 | PAPPA | CVD II | Q13219 | 0.2 |  |
| Proteinase-activated receptor 1 | PAR-1 | CVD II | P25116 | 0.0 |  |
| Poly [ADP-ribose] polymerase 1 | PARP-1 | CVD II | P09874 | 0.5 |  |
| Procollagen C-endopeptidase enhancer 1 | PCOLCE | CM | Q15113 | 0.0 |  |
| Platelet-derived growth factor subunit B | PDGF-SUBUNIT-B | CVD II | P01127 | 0.0 |  |
| Programmed cell death 1 ligand 2 | PD-L2 | CVD II | Q9BQ51 | 0.0 |  |
| Placenta growth factor | PGF | CVD II | P49763 | 0.0 |  |
| Polymeric immunoglobulin receptor | PIGR | CVD II | P01833 | 100.0 | Excluded |
| Platelet-activating factor acetylhydrolase | PLA2G7 | CM | Q13093 | 0.0 |  |
| Phospholipid transfer protein | PLTP | CM | P55058 | 0.7 |  |
| Plexin-B2 | PLXNB2 | CM | O15031 | 0.0 |  |
| Lysosomal Pro-X carboxypeptidase | PRCP | CM | P42785 | 49.6 |  |
| Prolargin | PRELP | CVD II | P51888 | 0.0 |  |
| Vitamin K-dependent protein C | PROC | CM | P04070 | 0.0 |  |
| Trypsin-2 | PRSS2 | CM | P07478 | 0.0 |  |
| Serine protease 27 | PRSS27 | CVD II | Q9BQR3 | 0.0 |  |
| Prostasin | PRSS8 | CVD II | Q16651 | 0.0 |  |
| P-selectin glycoprotein ligand 1 | PSGL-1 | CVD II | Q14242 | 0.0 |  |
| Receptor-type tyrosine-protein phosphatase S | PTPRS | CM | Q13332 | 0.0 |  |
| Pentraxin-related protein PTX3 | PTX3 | CVD II | P26022 | 0.1 |  |
| Glutaminyl-peptide cyclotransferase | QPCT | CM | Q16769 | 35.9 |  |
| Receptor for advanced glycosylation end products | RAGE | CVD II | Q15109 | 0.0 |  |
| Lithostathine-1-alpha | REG1A | CM | P05451 | 0.0 |  |
| Regenerating islet-derived protein 3-alpha | REG3A | CM | Q06141 | 98.3 | Excluded |
| Renin | REN | CVD II | P00797 | 0.0 |  |
| Serum amyloid A-4 protein | SAA4 | CM | P35542 | 0.0 |  |
| Stem cell factor | SCF | CVD II | P21583 | 0.0 |  |
| L-selectin | SELL | CM | P14151 | 0.0 |  |
| Serpin A12 | SERPINA12 | CVD II | Q8IW75 | 17.3 |  |
| Plasma serine protease inhibitor | SERPINA5 | CM | P05154 | 0.0 |  |
| Thyroxine-binding globulin | SERPINA7 | CM | P05543 | 0.0 |  |
| SLAM family member 7 | SLAMF7 | CVD II | Q9NQ25 | 0.2 |  |
| Superoxide dismutase [Cu-Zn] | SOD1 | CM | P00441 | 96.6 | Excluded |
| Superoxide dismutase [Mn], mitochondrial | SOD2 | CVD II | P04179 | 0.0 |  |
| Sortilin | SORT1 | CVD II | Q99523 | 0.0 |  |
| SPARC-like protein 1 | SPARCL1 | CM | Q14515 | 0.0 |  |
| Spondin-2 | SPON2 | CVD II | Q9BUD6 | 0.0 |  |
| Proto-oncogene tyrosine-protein kinase Src | SRC | CVD II | P12931 | 0.0 |  |
| Beta-galactoside alpha-2,6-sialyltransferase 1 | ST6GAL1 | CM | P15907 | 0.0 |  |
| Serine/threonine-protein kinase 4 | STK4 | CVD II | Q13043 | 4.4 |  |
| Transcobalamin-2 | TCN2 | CM | P20062 | 0.0 |  |
| Tissue factor | TF | CVD II | P13726 | 0.0 |  |
| Transforming growth factor-beta-induced protein ig-h3 | TGFBI | CM | Q15582 | 0.0 |  |
| Transforming growth factor beta receptor type 3 | TGFBR3 | CM | Q03167 | 11.9 |  |
| Protein-glutamine gamma-glutamyltransferase 2 | TGM2 | CVD II | P21980 | 0.0 |  |
| Thrombospondin-2 | THBS2 | CVD II | P35442 | 0.0 |  |
| Thrombospondin-4 | THBS4 | CM | P35443 | 0.0 |  |
| Thrombopoietin | THPO | CVD II | P40225 | 0.0 |  |
| Tyrosine-protein kinase receptor Tie-1 | TIE1 | CM | P35590 | 0.0 |  |
| Angiopoietin-1 receptor | TIE2 | CVD II | Q02763 | 0.0 |  |
| T-cell immunoglobulin and mucin domain-containing protein 4 | TIMD4 | CM | Q96H15 | 0.0 |  |
| Metalloproteinase inhibitor 1 | TIMP1 | CM | P01033 | 0.0 |  |
| Thrombomodulin TM | TM | CVD II | P07204 | 0.0 |  |
| Tenascin | TNC | CM | P24821 | 0.0 |  |
| Tumor necrosis factor receptor superfamily member 10A | TNFRSF10A | CVD II | O00220 | 0.0 |  |
| Tumor necrosis factor receptor superfamily member 11A | TNFRSF11A | CVD II | Q9Y6Q6 | 0.0 |  |
| Tumor necrosis factor receptor superfamily member 13B | TNFRSF13B | CVD II | O14836 | 0.0 |  |
| Tenascin-X | TNXB | CM | P22105 | 1.1 |  |
| TNF-related apoptosis-inducing ligand receptor 2 | TRAIL-R2 | CVD II | O14763 | 0.0 |  |
| Uromodulin | UMOD | CM | P07911 | 16.1 |  |
| Vasorin | VASN | CM | Q6EMK4 | 0.0 |  |
| Vascular cell adhesion protein 1 | VCAM1 | CM | P19320 | 0.0 |  |
| Vascular endothelial growth factor D | VEGFD | CVD II | O43915 | 0.0 |  |
| V-set and immunoglobulin domain-containing protein 2 | VSIG2 | CVD II | Q96IQ7 | 0.0 |  |
| Lymphotactin | XCL1 | CVD II | P47992 | 0.0 |  |
