# Supplementary material for: Heart failure in obesity: insights from proteomics in patients treated with or without weight-loss surgery
Source: Int J Obes (Lond). 2022 Aug 9;46(12):2088–94. doi: 10.1038/s41366-022-01194-0 (PMC9678794; doi:10.1038/s41366-022-01194-0)
Supplement: Supplementary file 4 — Supplmentary Table 1 [file 41366_2022_1194_MOESM4_ESM.docx]

### Supplementary Table 2: Association between proteins biomarkers and incident HF using logistic regression models adjusted for the matching variables (age, gender, bariatric surgery and duration of incident HF)

| **Protein name** | **Panel** | **OR (CI 95 %)** | **Standardized OR (CI 95 %)** | **p-value** | **FDR adjusted p-value** | **Proportion of significant**  **FDR adjusted p-values**  **(Bootstrap resampling)** |
| --- | --- | --- | --- | --- | --- | --- |
| TNFRSF10A | CVD II | 2.85 (1.86 - 4.37) | 1.44 (1.24 - 1.67) | <0.0001 | 0.0002 | 98.6 |
| ST6GAL1 | CM | 2.29 (1.55 - 3.37) | 1.36 (1.18 - 1.57) | <0.0001 | 0.002 | 93.2 |
| PRCP | CM | 3.26 (1.75 - 6.06) | 1.36 (1.16 - 1.60) | 0.0001 | 0.007 | 89.1 |
| MMP12 | CVD II | 1.53 (1.23 - 1.90) | 1.33 (1.15 - 1.54) | 0.0001 | 0.007 | 88.9 |
| TIMP1 | CM | 1.97 (1.38 - 2.81) | 1.32 (1.14 - 1.52) | 0.0001 | 0.007 | 86.4 |
| LPL | CVD II | 0.64 (0.50 - 0.82) | 0.76 (0.65 - 0.88) | 0.0003 | 0.008 | 85.9 |
| CCL3 | CVD II | 1.53 (1.21 - 1.93) | 1.31 (1.13 - 1.52) | 0.0004 | 0.008 | 85.2 |
| QPCT | CM | 3.37 (1.71 - 6.65) | 1.31 (1.13 - 1.53) | 0.0004 | 0.008 | 84.9 |
| ANG | CM | 1.77 (1.29 - 2.42) | 1.30 (1.12 - 1.50) | 0.0003 | 0.008 | 83.0 |
| C1QTNF1 | CM | 1.47 (1.18 - 1.82) | 1.30 (1.12 - 1.50) | 0.0004 | 0.008 | 82.4 |
| SERPINA5 | CM | 1.79 (1.28 - 2.50) | 1.28 (1.11 - 1.48) | 0.0007 | 0.009 | 80.1 |
| GAL-9 | CVD II | 2.19 (1.39 - 3.44) | 1.28 (1.11 - 1.48) | 0.0006 | 0.009 | 80.0 |
| KIM1 | CVD II | 1.37 (1.14 - 1.65) | 1.29 (1.11 - 1.50) | 0.0007 | 0.010 | 79.6 |
| COL18A1 | CM | 2.02 (1.35 - 3.04) | 1.28 (1.11 - 1.48) | 0.0006 | 0.009 | 79.4 |
| TRAIL-R2 | CVD II | 2.01 (1.33 - 3.03) | 1.28 (1.11 - 1.48) | 0.0008 | 0.010 | 77.9 |
| IL-1RA | CVD II | 1.36 (1.13 - 1.63) | 1.27 (1.10 - 1.47) | 0.0009 | 0.010 | 76.9 |
| ICAM1 | CM | 1.80 (1.27 - 2.56) | 1.27 (1.10 - 1.46) | 0.0009 | 0.010 | 75.4 |
| MMP7 | CVD II | 1.26 (1.09 - 1.46) | 1.25 (1.08 - 1.44) | 0.002 | 0.020 | 70.8 |
| IL6 | CVD II | 1.35 (1.11 - 1.63) | 1.25 (1.09 - 1.45) | 0.002 | 0.020 | 70.4 |
| FGF-21 | CVD II | 1.20 (1.07 - 1.35) | 1.24 (1.08 - 1.43) | 0.002 | 0.021 | 69.9 |
| CES1 | CM | 1.25 (1.08 - 1.44) | 1.24 (1.08 - 1.43) | 0.003 | 0.024 | 68.4 |
| SCF | CVD II | 0.64 (0.48 - 0.86) | 0.80 (0.70 - 0.93) | 0.003 | 0.024 | 68.2 |
| PRSS8 | CVD II | 1.77 (1.21 - 2.60) | 1.25 (1.08 - 1.44) | 0.003 | 0.024 | 65.6 |
| CST3 | CM | 1.69 (1.19 - 2.40) | 1.24 (1.07 - 1.42) | 0.003 | 0.024 | 65.3 |
| LILRB1 | CM | 1.82 (1.22 - 2.72) | 1.23 (1.07 - 1.42) | 0.003 | 0.025 | 64.7 |
| REG1A | CM | 1.46 (1.12 - 1.89) | 1.23 (1.07 - 1.41) | 0.004 | 0.029 | 62.7 |
| NID1 | CM | 1.65 (1.17 - 2.34) | 1.23 (1.06 - 1.41) | 0.004 | 0.029 | 62.6 |
| C2 | CM | 1.65 (1.17 - 2.32) | 1.23 (1.07 - 1.42) | 0.004 | 0.029 | 62.5 |
| CFHR5 | CM | 1.54 (1.14 - 2.10) | 1.22 (1.06 - 1.41) | 0.005 | 0.033 | 61.2 |
| IGLC2 | CM | 1.45 (1.11 - 1.90) | 1.21 (1.06 - 1.40) | 0.006 | 0.038 | 57.8 |
| TCN2 | CM | 1.61 (1.13 - 2.29) | 1.21 (1.05 - 1.39) | 0.008 | 0.046 | 55.3 |
| TIE1 | CM | 1.99 (1.19 - 3.31) | 1.21 (1.05 - 1.39) | 0.008 | 0.046 | 55.2 |
| AOC3 | CM | 1.64 (1.12 - 2.41) | 1.20 (1.04 - 1.38) | 0.011 | 0.060 | 52.4 |
| GIF | CVD II | 1.25 (1.05 - 1.48) | 1.20 (1.04 - 1.38) | 0.011 | 0.060 | 51.7 |
| VEGFD | CVD II | 1.49 (1.09 - 2.03) | 1.21 (1.04 - 1.40) | 0.011 | 0.060 | 51.1 |
| CCL18 | CM | 1.26 (1.05 - 1.51) | 1.20 (1.04 - 1.38) | 0.012 | 0.063 | 49.8 |
| LOX-1 | CVD II | 1.29 (1.05 - 1.58) | 1.19 (1.04 - 1.37) | 0.014 | 0.066 | 49.3 |
| TNC | CM | 1.36 (1.07 - 1.74) | 1.19 (1.04 - 1.37) | 0.013 | 0.064 | 48.0 |
| PSGL-1 | CVD II | 1.78 (1.11 - 2.86) | 1.19 (1.03 - 1.38) | 0.016 | 0.072 | 47.9 |
| PRSS2 | CM | 1.37 (1.06 - 1.77) | 1.19 (1.03 - 1.37) | 0.016 | 0.072 | 46.8 |
| CCL17 | CVD II | 1.17 (1.03 - 1.32) | 1.18 (1.03 - 1.36) | 0.016 | 0.072 | 46.0 |
| SPON2 | CVD II | 2.05 (1.12 - 3.74) | 1.18 (1.03 - 1.36) | 0.019 | 0.083 | 45.1 |
| CCL14 | CM | 1.49 (1.05 - 2.09) | 1.18 (1.02 - 1.35) | 0.023 | 0.096 | 41.9 |
| THBS2 | CVD II | 1.97 (1.09 - 3.58) | 1.17 (1.02 - 1.35) | 0.025 | 0.099 | 40.8 |
| CDH1 | CM | 1.48 (1.05 - 2.09) | 1.17 (1.02 - 1.35) | 0.026 | 0.10 | 40.1 |
| PRSS27 | CVD II | 1.42 (1.05 - 1.92) | 1.17 (1.02 - 1.35) | 0.023 | 0.096 | 39.8 |
| FCGR3B | CM | 1.33 (1.03 - 1.73) | 1.17 (1.02 - 1.34) | 0.028 | 0.10 | 38.2 |
| LCN2 | CM | 2.13 (1.07 - 4.24) | 1.17 (1.01 - 1.35) | 0.031 | 0.11 | 36.5 |
| OSMR | CM | 2.00 (1.03 - 3.90) | 1.16 (1.01 - 1.33) | 0.041 | 0.13 | 34.9 |
| CNDP1 | CM | 1.30 (1.02 - 1.66) | 1.16 (1.01 - 1.34) | 0.036 | 0.13 | 34.8 |
| ANGPTL3 | CM | 1.46 (1.02 - 2.10) | 1.16 (1.01 - 1.33) | 0.041 | 0.13 | 34.5 |
| CTSL1 | CVD II | 1.59 (1.03 - 2.46) | 1.16 (1.01 - 1.34) | 0.038 | 0.13 | 34.2 |
| TGFBI | CM | 1.39 (1.01 - 1.91) | 1.16 (1.01 - 1.33) | 0.040 | 0.13 | 33.8 |
| FCN2 | CM | 1.32 (1.01 - 1.74) | 1.16 (1.00 - 1.33) | 0.044 | 0.14 | 32.8 |
| GAS6 | CM | 1.45 (1.00 - 2.10) | 1.15 (1.00 - 1.32) | 0.049 | 0.15 | 31.9 |
| AMBP | CVD II | 2.06 (1.02 - 4.16) | 1.15 (1.00 - 1.33) | 0.045 | 0.14 | 31.8 |
| CD40-L | CVD II | 1.09 (1.00 - 1.19) | 1.15 (1.00 - 1.32) | 0.049 | 0.15 | 31.1 |
| CCL5 | CM | 1.13 (1.00 - 1.28) | 1.15 (1.00 - 1.32) | 0.052 | 0.15 | 30.8 |
| F11 | CM | 1.35 (1.00 - 1.84) | 1.15 (1.00 - 1.32) | 0.052 | 0.15 | 30.7 |
| GLO1 | CVD II | 1.13 (1.00 - 1.27) | 1.15 (1.00 - 1.32) | 0.053 | 0.15 | 30.1 |
| CA5A | CVD II | 1.16 (1.00 - 1.35) | 1.15 (1.00 - 1.33) | 0.052 | 0.15 | 29.7 |
| PLA2G7 | CM | 1.48 (0.99 - 2.20) | 1.15 (1.00 - 1.33) | 0.054 | 0.15 | 29.7 |
| VCAM1 | CM | 1.42 (0.99 - 2.06) | 1.14 (0.99 - 1.31) | 0.059 | 0.16 | 29.5 |
| MARCO | CVD II | 1.67 (0.97 - 2.85) | 1.14 (0.99 - 1.32) | 0.062 | 0.17 | 29.1 |
| FS | CVD II | 1.32 (0.98 - 1.78) | 1.14 (0.99 - 1.31) | 0.063 | 0.17 | 28.3 |
| CEACAM8 | CVD II | 1.26 (0.99 - 1.61) | 1.14 (0.99 - 1.31) | 0.064 | 0.17 | 28.1 |
| IL18 | CVD II | 1.27 (0.98 - 1.64) | 1.14 (0.99 - 1.31) | 0.071 | 0.18 | 27.3 |
| IL-4RA | CVD II | 1.47 (0.98 - 2.21) | 1.14 (0.99 - 1.31) | 0.063 | 0.17 | 27.2 |
| NEMO | CVD II | 1.09 (0.99 - 1.20) | 1.14 (0.99 - 1.31) | 0.067 | 0.17 | 26.8 |
| CD46 | CM | 1.43 (0.96 - 2.12) | 1.13 (0.99 - 1.30) | 0.075 | 0.18 | 26.7 |
| PAM | CM | 1.50 (0.97 - 2.33) | 1.14 (0.99 - 1.30) | 0.069 | 0.18 | 26.3 |
| CD4 | CVD II | 1.39 (0.97 - 2.00) | 1.14 (0.99 - 1.31) | 0.075 | 0.18 | 26.0 |
| DECR1 | CVD II | 1.08 (0.99 - 1.17) | 1.13 (0.99 - 1.30) | 0.076 | 0.18 | 25.3 |
| SORT1 | CVD II | 1.38 (0.96 - 2.00) | 1.13 (0.98 - 1.30) | 0.084 | 0.19 | 25.0 |
| FABP2 | CVD II | 1.19 (0.98 - 1.44) | 1.13 (0.98 - 1.30) | 0.081 | 0.19 | 24.6 |
| HOSCAR | CVD II | 1.62 (0.94 - 2.80) | 1.13 (0.98 - 1.30) | 0.083 | 0.19 | 23.9 |
| PLXNB2 | CM | 1.35 (0.95 - 1.90) | 1.13 (0.98 - 1.29) | 0.092 | 0.20 | 23.1 |
| DKK-1 | CVD II | 1.16 (0.98 - 1.39) | 1.13 (0.98 - 1.30) | 0.086 | 0.19 | 23.0 |
| ACE2 | CVD II | 1.18 (0.98 - 1.43) | 1.14 (0.98 - 1.31) | 0.082 | 0.19 | 22.9 |
| PROC | CM | 1.32 (0.95 - 1.85) | 1.12 (0.98 - 1.29) | 0.10 | 0.21 | 21.8 |
| HSP-27 | CVD II | 1.17 (0.97 - 1.40) | 1.12 (0.98 - 1.29) | 0.099 | 0.21 | 21.1 |
| ICAM3 | CM | 1.38 (0.93 - 2.04) | 1.12 (0.98 - 1.29) | 0.10 | 0.22 | 20.5 |
| IGG-FC-RECEPTOR-II-B | CVD II | 1.16 (0.97 - 1.39) | 1.12 (0.98 - 1.29) | 0.10 | 0.21 | 20.5 |
| PAPPA | CVD II | 0.80 (0.62 - 1.03) | 0.88 (0.76 - 1.02) | 0.087 | 0.19 | 19.8 |
| SAA4 | CM | 1.21 (0.95 - 1.55) | 1.11 (0.97 - 1.28) | 0.12 | 0.26 | 18.8 |
| PDGF-SUBUNIT-B | CVD II | 1.11 (0.97 - 1.26) | 1.12 (0.97 - 1.28) | 0.12 | 0.25 | 18.8 |
| ADM | CVD II | 1.26 (0.93 - 1.71) | 1.11 (0.97 - 1.28) | 0.13 | 0.27 | 18.0 |
| STK4 | CVD II | 1.07 (0.98 - 1.16) | 1.11 (0.97 - 1.28) | 0.12 | 0.26 | 17.9 |
| LILRB2 | CM | 1.32 (0.92 - 1.89) | 1.11 (0.97 - 1.28) | 0.13 | 0.26 | 17.7 |
| CD59 | CM | 1.54 (0.84 - 2.83) | 1.11 (0.96 - 1.28) | 0.16 | 0.30 | 17.6 |
| PCOLCE | CM | 1.27 (0.93 - 1.75) | 1.11 (0.97 - 1.28) | 0.13 | 0.26 | 17.2 |
| FETUB | CM | 1.26 (0.93 - 1.70) | 1.11 (0.97 - 1.28) | 0.13 | 0.27 | 17.1 |
| HB-EGF | CVD II | 1.13 (0.96 - 1.33) | 1.11 (0.96 - 1.27) | 0.15 | 0.28 | 16.7 |
| SRC | CVD II | 1.08 (0.97 - 1.19) | 1.11 (0.96 - 1.27) | 0.14 | 0.28 | 16.4 |
| ANG-1 | CVD II | 1.11 (0.96 - 1.28) | 1.10 (0.96 - 1.27) | 0.15 | 0.29 | 15.5 |
| REN | CVD II | 1.14 (0.94 - 1.38) | 1.10 (0.96 - 1.27) | 0.17 | 0.31 | 14.7 |
| TNFRSF11A | CVD II | 1.24 (0.90 - 1.70) | 1.10 (0.96 - 1.26) | 0.18 | 0.33 | 14.7 |
| F7 | CM | 1.25 (0.89 - 1.76) | 1.10 (0.95 - 1.26) | 0.19 | 0.33 | 14.5 |
| MEGF9 | CM | 1.29 (0.88 - 1.87) | 1.10 (0.96 - 1.26) | 0.18 | 0.33 | 14.2 |
| HAOX1 | CVD II | 1.07 (0.97 - 1.19) | 1.10 (0.96 - 1.27) | 0.16 | 0.31 | 13.9 |
| AGRP | CVD II | 1.22 (0.91 - 1.65) | 1.10 (0.96 - 1.27) | 0.18 | 0.33 | 13.8 |
| VSIG2 | CVD II | 1.21 (0.90 - 1.62) | 1.10 (0.95 - 1.26) | 0.20 | 0.33 | 13.3 |
| ITGAM | CM | 1.43 (0.83 - 2.48) | 1.09 (0.95 - 1.26) | 0.20 | 0.33 | 12.3 |
| GT | CVD II | 1.17 (0.92 - 1.49) | 1.10 (0.95 - 1.26) | 0.19 | 0.33 | 11.9 |
| TNFRSF13B | CVD II | 1.25 (0.87 - 1.80) | 1.09 (0.95 - 1.25) | 0.22 | 0.35 | 11.8 |
| FGF-23 | CVD II | 1.14 (0.93 - 1.41) | 1.09 (0.95 - 1.26) | 0.21 | 0.35 | 11.5 |
| LEP | CVD II | 1.16 (0.91 - 1.49) | 1.12 (0.93 - 1.34) | 0.23 | 0.36 | 10.8 |
| EFEMP1 | CM | 1.23 (0.87 - 1.73) | 1.09 (0.94 - 1.26) | 0.24 | 0.37 | 10.7 |
| CR2 | CM | 1.16 (0.90 - 1.50) | 1.08 (0.94 - 1.24) | 0.26 | 0.40 | 10.7 |
| SERPINA12 | CVD II | 0.90 (0.77 - 1.06) | 0.91 (0.80 - 1.05) | 0.20 | 0.34 | 10.7 |
| PTX3 | CVD II | 1.17 (0.88 - 1.55) | 1.08 (0.94 - 1.24) | 0.26 | 0.41 | 10.3 |
| BOC | CVD II | 0.73 (0.45 - 1.17) | 0.91 (0.79 - 1.05) | 0.18 | 0.33 | 10.2 |
| SERPINA7 | CM | 1.21 (0.85 - 1.72) | 1.08 (0.94 - 1.24) | 0.29 | 0.43 | 10.2 |
| IDUA | CVD II | 1.16 (0.90 - 1.49) | 1.09 (0.95 - 1.25) | 0.24 | 0.37 | 10.2 |
| MFAP5 | CM | 1.26 (0.83 - 1.91) | 1.08 (0.94 - 1.24) | 0.28 | 0.41 | 9.4 |
| NCAM1 | CM | 0.78 (0.52 - 1.16) | 0.91 (0.79 - 1.05) | 0.21 | 0.35 | 9.2 |
| TGFBR3 | CM | 0.86 (0.68 - 1.10) | 0.92 (0.80 - 1.05) | 0.22 | 0.35 | 9.2 |
| PGF | CVD II | 1.26 (0.80 - 1.99) | 1.08 (0.93 - 1.24) | 0.31 | 0.45 | 8.9 |
| TF | CVD II | 0.75 (0.49 - 1.16) | 0.91 (0.79 - 1.05) | 0.20 | 0.33 | 8.8 |
| UMOD | CM | 0.74 (0.46 - 1.20) | 0.92 (0.80 - 1.05) | 0.22 | 0.35 | 8.8 |
| MET | CM | 1.27 (0.81 - 2.00) | 1.08 (0.94 - 1.23) | 0.30 | 0.43 | 8.7 |
| TGM2 | CVD II | 1.08 (0.94 - 1.25) | 1.08 (0.94 - 1.24) | 0.27 | 0.41 | 8.7 |
| PARP-1 | CVD II | 1.10 (0.90 - 1.35) | 1.07 (0.93 - 1.23) | 0.34 | 0.47 | 8.1 |
| CA4 | CM | 1.20 (0.80 - 1.81) | 1.07 (0.93 - 1.22) | 0.36 | 0.47 | 8.0 |
| GNLY | CM | 1.22 (0.81 - 1.86) | 1.07 (0.93 - 1.23) | 0.34 | 0.47 | 7.8 |
| ITGB1BP2 | CVD II | 1.04 (0.96 - 1.14) | 1.07 (0.93 - 1.23) | 0.34 | 0.47 | 7.7 |
| CRTAC1 | CM | 0.88 (0.70 - 1.11) | 0.93 (0.81 - 1.06) | 0.28 | 0.41 | 7.6 |
| IGFBP6 | CM | 1.20 (0.81 - 1.76) | 1.07 (0.93 - 1.23) | 0.36 | 0.47 | 7.5 |
| TIE2 | CVD II | 1.26 (0.77 - 2.08) | 1.07 (0.93 - 1.23) | 0.36 | 0.47 | 7.4 |
| IGFBP3 | CM | 1.15 (0.87 - 1.53) | 1.07 (0.93 - 1.23) | 0.33 | 0.47 | 7.2 |
| CXCL1 | CVD II | 1.06 (0.94 - 1.21) | 1.07 (0.93 - 1.23) | 0.33 | 0.47 | 7.2 |
| SLAMF7 | CVD II | 1.11 (0.89 - 1.38) | 1.07 (0.93 - 1.23) | 0.35 | 0.47 | 7.1 |
| CA3 | CM | 1.12 (0.88 - 1.43) | 1.07 (0.93 - 1.22) | 0.36 | 0.47 | 7.0 |
| PAR-1 | CVD II | 1.10 (0.89 - 1.38) | 1.06 (0.93 - 1.22) | 0.37 | 0.48 | 6.9 |
| APOM | CM | 1.16 (0.80 - 1.71) | 1.06 (0.92 - 1.21) | 0.43 | 0.52 | 6.8 |
| PRELP | CVD II | 1.35 (0.68 - 2.66) | 1.06 (0.92 - 1.23) | 0.39 | 0.49 | 6.6 |
| ENG | CM | 1.24 (0.75 - 2.06) | 1.06 (0.92 - 1.22) | 0.40 | 0.50 | 6.6 |
| TIMD4 | CM | 1.12 (0.87 - 1.46) | 1.06 (0.92 - 1.23) | 0.38 | 0.49 | 6.6 |
| NRP1 | CM | 1.31 (0.69 - 2.49) | 1.06 (0.92 - 1.22) | 0.41 | 0.51 | 6.5 |
| HO-1 | CVD II | 0.86 (0.64 - 1.15) | 0.93 (0.81 - 1.07) | 0.29 | 0.43 | 6.5 |
| IL16 | CVD II | 1.12 (0.85 - 1.49) | 1.06 (0.92 - 1.22) | 0.41 | 0.52 | 6.0 |
| DPP4 | CM | 1.16 (0.81 - 1.66) | 1.06 (0.92 - 1.22) | 0.42 | 0.52 | 5.6 |
| PTPRS | CM | 0.78 (0.45 - 1.33) | 0.94 (0.82 - 1.08) | 0.35 | 0.47 | 5.3 |
| IL1RL2 | CVD II | 1.15 (0.83 - 1.59) | 1.06 (0.92 - 1.22) | 0.39 | 0.49 | 5.2 |
| MERTK | CVD II | 1.16 (0.80 - 1.67) | 1.06 (0.92 - 1.22) | 0.43 | 0.52 | 5.2 |
| SPARCL1 | CM | 0.84 (0.59 - 1.20) | 0.94 (0.82 - 1.07) | 0.34 | 0.47 | 4.6 |
| CA1 | CM | 1.05 (0.93 - 1.19) | 1.05 (0.92 - 1.21) | 0.45 | 0.54 | 4.4 |
| TM | CVD II | 1.13 (0.74 - 1.72) | 1.04 (0.91 - 1.20) | 0.56 | 0.67 | 4.3 |
| PLTP | CM | 0.85 (0.57 - 1.29) | 0.95 (0.82 - 1.09) | 0.44 | 0.54 | 3.5 |
| SOD2 | CVD II | 1.34 (0.43 - 4.16) | 1.04 (0.90 - 1.19) | 0.61 | 0.70 | 3.5 |
| GP1BA | CM | 1.08 (0.82 - 1.42) | 1.04 (0.90 - 1.19) | 0.59 | 0.69 | 3.4 |
| NOTCH1 | CM | 1.12 (0.72 - 1.76) | 1.04 (0.90 - 1.19) | 0.60 | 0.70 | 3.2 |
| RAGE | CVD II | 0.92 (0.66 - 1.27) | 0.96 (0.84 - 1.11) | 0.59 | 0.69 | 3.1 |
| FAP | CM | 0.84 (0.44 - 1.59) | 0.96 (0.84 - 1.10) | 0.58 | 0.68 | 2.6 |
| COMP | CM | 0.90 (0.66 - 1.24) | 0.96 (0.83 - 1.10) | 0.53 | 0.63 | 2.6 |
| ADAM-TS13 | CVD II | 0.87 (0.50 - 1.51) | 0.97 (0.84 - 1.11) | 0.62 | 0.71 | 2.4 |
| CHL1 | CM | 1.06 (0.71 - 1.57) | 1.02 (0.89 - 1.17) | 0.77 | 0.85 | 2.4 |
| THPO | CVD II | 1.04 (0.72 - 1.51) | 1.01 (0.88 - 1.17) | 0.83 | 0.89 | 2.4 |
| GDF-2 | CVD II | 1.05 (0.79 - 1.40) | 1.02 (0.89 - 1.18) | 0.74 | 0.82 | 2.3 |
| IL-17D | CVD II | 1.09 (0.72 - 1.66) | 1.03 (0.89 - 1.19) | 0.67 | 0.76 | 2.2 |
| KIT | CM | 0.94 (0.66 - 1.34) | 0.98 (0.85 - 1.12) | 0.74 | 0.82 | 2.0 |
| THBS4 | CM | 1.02 (0.80 - 1.29) | 1.01 (0.88 - 1.16) | 0.88 | 0.92 | 1.9 |
| FCGR2A | CM | 1.01 (0.80 - 1.29) | 1.01 (0.88 - 1.16) | 0.91 | 0.94 | 1.8 |
| IL7R | CM | 0.96 (0.74 - 1.25) | 0.98 (0.85 - 1.12) | 0.76 | 0.84 | 1.7 |
| BMP-6 | CVD II | 1.02 (0.87 - 1.19) | 1.01 (0.88 - 1.17) | 0.83 | 0.89 | 1.6 |
| CD84 | CVD II | 1.00 (0.79 - 1.26) | 1.00 (0.87 - 1.15) | 0.99 | 0.99 | 1.5 |
| PD-L2 | CVD II | 0.98 (0.69 - 1.41) | 0.99 (0.87 - 1.14) | 0.93 | 0.94 | 1.5 |
| XCL1 | CVD II | 1.01 (0.81 - 1.27) | 1.01 (0.88 - 1.16) | 0.90 | 0.94 | 1.5 |
| SELL | CM | 0.94 (0.64 - 1.36) | 0.98 (0.85 - 1.12) | 0.72 | 0.81 | 1.4 |
| LILRB5 | CM | 0.98 (0.82 - 1.17) | 0.98 (0.86 - 1.13) | 0.78 | 0.85 | 1.4 |
| CTRC | CVD II | 0.98 (0.82 - 1.17) | 0.98 (0.86 - 1.13) | 0.80 | 0.86 | 1.4 |
| DCN | CVD II | 1.02 (0.60 - 1.76) | 1.01 (0.87 - 1.16) | 0.93 | 0.94 | 1.4 |
| IL-27 | CVD II | 1.01 (0.73 - 1.42) | 1.01 (0.88 - 1.15) | 0.93 | 0.94 | 1.4 |
| GH | CVD II | 1.00 (0.93 - 1.08) | 1.00 (0.87 - 1.16) | 0.96 | 0.96 | 1.3 |
| VASN | CM | 0.92 (0.57 - 1.47) | 0.97 (0.85 - 1.12) | 0.71 | 0.80 | 1.3 |
| MBL2 | CM | 1.01 (0.90 - 1.13) | 1.01 (0.88 - 1.16) | 0.90 | 0.93 | 1.3 |
| LYVE1 | CM | 0.97 (0.67 - 1.39) | 0.99 (0.86 - 1.13) | 0.85 | 0.90 | 1.3 |
| TNXB | CM | 0.95 (0.54 - 1.69) | 0.99 (0.86 - 1.13) | 0.86 | 0.91 | 1.2 |

CI = confidence interval; CM = Cardiometabolic; CVD II = Cardiovascular II; FDR = false discovery rate; HF = heart failure; OR = odds ratio.
